# Supplementary material for: Learning curve and surgical outcome of robotic assisted colorectal surgery with ERAS program
Source: Sci Rep. 2022 Nov 29;12:20566. doi: 10.1038/s41598-022-24665-w (PMC9709162; doi:10.1038/s41598-022-24665-w)
Supplement: Supplementary file 2 — Supplementary Information 2. [file 41598_2022_24665_MOESM2_ESM.zip › RAL Raw data-Ñ[▒K/075/OP N_ñw╝╨░O▒Kñσ.pdf]

W95 / W95

臺中榮民總醫院
手術室護理記錄

[redacted]

基本資料

- 室別： 開刀房-17室，手術科別： CRS，手術類別： 常規，日期： 2021/04/22
- 進入時間： 0730 ； 麻醉開始時間： 0735 ； Time Out時間： 0910 ； 劃刀時間： 0911 ； 手術結束時間： 1355 送出時間： 1405 ；
- 麻醉方式： GE
  診斷： Malignant tumor of rectum
  手術名稱： Robot Assisted Low Anterior Resection
  手術局部用藥： nil
  術前預防性抗生素：
  - 抗生素名稱一： Veterin 1GM IVD由麻醉護理人員執行
  - 時間： 0850
  - 上次簽章: 陳祐溥
  ，追加預防性抗生素：
- 手術醫師： 蔣鋒帆，楊曙榮，陳勇先
- 麻醉醫師： VS 齊光彪
-
  - 刷手護士A ： 吳貞儀，時間： 0730，刷手護士B ： 陳盈妤，時間： 0730，接替刷手一： 陳祐溥，時間： 1300
  - 流動護士A ： 陳祐溥，時間： 0730，接替流動一： 陳盈妤，時間： 1100-1200，接替流動二： 吳貞儀，時間： 1300

皮膚狀況

A-V Shunt： 無
術前： 完整
記錄者： 上次簽章: 陳祐溥
術後： 完整
記錄者： 上次簽章: 吳貞儀

手術準備

手術臥式 單一手術臥式

-
  - 手術臥式： 仰臥
  - 使用附件： 矽膠枕頭，腳架，其它： 海綿墊
  - 皮膚準備： 10%Aq Betadine，Hibiscrub，2% Chlorhexidine
  - 電燒： 使用電燒: 電極片位置： 右大腿，雙極電燒，記錄者： 上次簽章: 陳祐溥

止血帶T： 無

植入物

記錄者：

標本送檢

- 細菌培養 無
- 細胞學 無
- 冰凍切片 無
- 病理標本
  組織名稱一：Rectum
  ,
  組織名稱二：Distal cut end
  ,
  組織名稱三：IMA Root
  ，更多組織名稱
  - 組織名稱四：pelvic
  -

- ○ 組織名稱五： paraortic LN
- 截肢 無

標本交付 標本： 3 袋 ， 標本： 0 盒 ， 標本： 2 瓶 ， 細胞學： 0 管 ， Culture Tube： 0 管 ， 被交付醫師： R 陳勇先 ，

- 記錄者： 上次簽章: 吳貞儀
- 時間： 1330

傷口術後敷料

傷口敷料： 傷口敷料： 無

- 請於圖上標示部位：

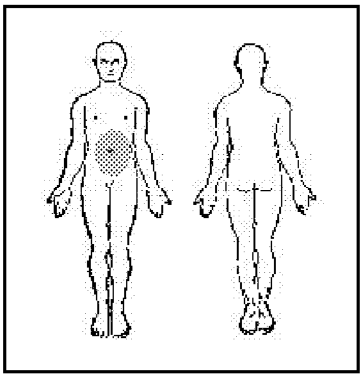

- 傷口敷料： 柔軟敷料覆蓋

導管

導管	天數	導管資訊	插入日期	插入時間	更換日期	拔除日期	拔除時間	拔除原因	預計拔除/更換	預計拔除/更換日期
Epidural PCA(1)	1	部位:脊髓;	2021/04/22	0750						
Sump NG Tube(1)	1	號碼:16 Fr.; 部位:胃;	2021/04/22	0755						
Rod,Stoma Supporting (1)	1	部位:小腸;	2021/04/22	1325						
Double Lumen CVC(1)	1	號碼:8 Fr.; 深度:fix 14 cm; 部位:jugular (右); 象限:RU;	2021/04/22	0845						
A-line(1)	1	號碼:20 G; 部位:左手;	2021/04/22	0850						
Foley(1)	1	號碼:16 Fr.; 部位:膀胱;	2021/04/22	0758						

用物計數

- 手術中出血量： 150 c.c.
-
  - ○ 其它： 無
  - ○ NIL

用物計數： 多切口同時縫合

- 1.器械： 正確 ，2.縫針： 正確 ，3.刀片 ： 正確 ，4.紗布： 正確 ，5.腹部墊： 未使用 ，6.紗球： 未使用 ，7.PEANUTS ： 未使用 ，8.紗條： 未使用 ，9.絲帶： 未使用 ，10.針頭： 未使用 ，11.Vessel loop： 未使用 ，12.Boots： 未使用 ，13.Jelco： 未使用 ，14.棉條： 未使用 ，
  - ○ 第一次：
    - ■ 流動護士：

，
  - ○ 第二次：
    - ■ 流動護士：

，
  - ○ 第三次：
    - ■ 流動護士：

備註

備註： 使用達文西XI 30度 NO.2。

- 記錄者： 上次簽章: 陳祐嫥
- 時間： 0745

填寫護理人員_吳貞儀

正式摘要

W95 / W95

doctorRank:B
